# Mandatory certification for clozapine prescribing

**DOI:** 10.1192/j.eurpsy.2020.110

**Published:** 2020-12-21

**Authors:** Dan Cohen, Saeed Farooq

**Affiliations:** 1 MHO North-Holland North, Heerhugowaard, The Netherlands; 2 School of Medicine, Keele University, Staffordshire.

**Keywords:** Certification program, clozapine, mandatory training, prescription barriers

## The Clozapine Prescription Rates and Current Clinical Practice

Clozapine is the only registered treatment for therapy-resistant schizophrenia (meaning, after treatment with two other antipsychotics failed or were not tolerated), psychosis in Parkinson’s disease, and, in the United States, for suicidality. Off-label, clozapine is prescribed for therapy-resistant disorders, especially therapy-resistant bipolar, borderline personality, violent behavior, or self-harm in patients with psychosis, aggressive behavior, or addiction to cannabis in psychotic disorder and in the animal model.

Despite the good evidence of efficacy [1] and now robust evidence that clozapine use may be associated with lowered mortality compared to other antipsychotics [2], the use of clozapine remains suboptimal in most countries, both within and outside Europe [3].

Low prescription rates not only result in poor treatment for schizophrenia, an unintended consequence is that trainees and consultants in psychiatry have less experience and, therefore, lack confidence in initiating and maintaining the clozapine therapy. In a major survey of UK practice, it was found that almost one third of senior consultants have not prescribed the clozapine, for more than a year. It appears that a vicious cycle sets in; because clozapine is less prescribed, doctors have less exposure and confidence in prescribing it which in turn results in even lesser exposure to managing the drug and its side effects. Not surprisingly, this promotes the aura for clozapine as a dangerous drug, which results in prescribers’ fear.

## Educational Interventions for Improving the Clinical Practice in Treatment-Resistant Schizophrenia

Systematic reviews on barriers to effective clozapine use identify lack of adequate training in initiating or maintaining clozapine use as a major barrier [4]. Inadequate knowledge or experience in clozapine use, fear of side effects, lack of knowledge about clozapine side effects, lack of adherence with guidance and difficulty identifying suitable patients, and unclear guidance regarding clozapine monitoring were identified as a major contributor to poor prescribing for clozapine by majority of participants in these studies.

Many psychiatry training programs do not include specific aspects of clozapine initiation and monitoring in the syllabi. In the United Kingdom, for example, the Royal College of Psychiatrists, the core training in psychiatry only mentions treatment-resistant schizophrenia management as a learning objective, without any mention of clozapine. In the higher training in general adult psychiatry, there is only one learning objective (intended learning outcome [ILO]) related to treatment of severe mental illness [5]. Mostly, the textbooks in psychiatry cover the risks associated with clozapine use and monitoring, but the evidence related to beneficial effects of clozapine and proactive approach in managing side effects is rarely covered. While the emphasis on monitoring clozapine use is understandable, this merely reinforces the clozapine as a dangerous drug.

There is some evidence that educational interventions improve the rates and quality of clozapine prescriptions. These educational interventions include Dutch [6], the United States [7], Australia [8], and New Zealand [9] examples. The content of these educational interventions varies but essentially covers following aspects: increased training in clozapine in identifying suitable patients for clozapine, education in side effects management, developing and implementing standardized protocols for medical review, supporting documentation and adverse event management, shared decision-making, and training in use of point care devices.

However laudable and effective these interventions are, they remain isolated efforts, which are not supported by educational institutions. The content and methodology of these training programs are also not standardized.

## A Mandatory Training and Certification Program for Clozapine

We propose a mandatory training program that requires a certification in core competencies for initiating and maintaining clozapine in psychiatry residence programs. Clozapine is not a commonly used drug in psychiatric practice, and trainees are less likely to be exposed to using clozapine prescription and management. The requirements for mandatory monitoring, potential benefits from effective use and wide array of side effects and the expertise needed for their management requires a mandatory program of training and certification in clozapine use.

This will ensure all trainees getting uniform training in essential clozapine prescribing. This will also provide confidence to service users. The syllabus, ILOs, and the assessment methods for such a training program can be constructed after consultations with the experts in the field. Number of skills and competencies in medicine and psychiatry require certain standards to be met.

In psychiatry, for example, minimum standards of training are required for practitioners of electroconvulsive therapy (ECT). The good practice guide to ECT training in the United Kingdom requires that junior trainees should be able to administer ECT without direct supervision, prepare patients for ECT, and explain to patients and relatives the facts about ECT, its indications, and broad place within psychiatric treatment. They ought to be able to monitor a patient’s mental state and cognitive functioning during a course of ECT. It is further specified that the higher trainees ought to maintain this capability until the end of year 6. The good practice guide also elaborates the details of competencies in theory and practice of ECT that must be met in order for a practitioner to administer ECT [10].

We suggest that a similar training program for clozapine initiation and maintaining must become part of basic and higher psychiatric training programs. Based on our research in this field and delivering workshops for effective clozapine prescriptions in the EPA congress and other forums, we suggest following broad areas covered in such a training program.

### Clozapine prescription

Indications—The training must include both registered and off-label indications of clozapine use. The evidence for efficacy and effectiveness, strategies for clozapine initiation, titration, and in maintenance therapy, audit, and monitoring in clinical practice will also be included in training programme.

## Monitoring and Safety Strategies and Procedures

### Side effects

The incidence and ways of early detection of the four main rare, but potential lethal, side effects: agranulocytosis, diabetic ketoacidosis, ileus, and myocarditis. The incidence and detection of common harmless but bothersome common side effects, such as sialorrhea and constipation. The treatment strategies for these side effects.

### Interactions

The liver cytochrome system (CYP) inducers, such as carbamazepine and the polyaromatic compounds of tobacco smoke, and with CYP inhibitors, such as caffeine (in coffee, cola, or many energy drinks), psychiatric, and nonpsychiatric (co-)medication.

### POCT

The availability, possibilities, and use of point-of-care devices.

### Involving service users in shared decision-making

This should include principles of shared decision-making (SDM) in initiating and maintaining clozapine treatment, support for patients and families, decision aid tools to assist SDM, capacity and consent for starting clozapine use, and internet-based educational programs to provide information for consumers, family members, and clinicians, involving service users in monitoring for safety.

The assessment methods for these training programs may include Multiple Choice Questions (MCQs), patient scenarios, and objective structured clinical examination for service users involvement. The syllabus, learning methods, and evaluation of learning outcomes for such a program can be developed in consultation with psychiatry residency programs across Europe in collaboration with EPA.

The ILO for such a program can be developed for core psychiatry training and higher psychiatric programs in consultation with the psychiatric training program in different countries. The essential elements of such programs can be developed by the EPA Committee on Education.

## Conclusion

A structural approach of professionals’ barriers to clozapine prescription requires adequate and sufficient education of both psychiatrists and trainees on all aspects of clozapine.

The logical consequence is that education and training in the use of clozapine must become a mandatory requirement of all psychiatry residence and Continuing Professional Development (CPD) programs, similar to the certification for ECT.
